# An atlas of cortical arealization identifies dynamic molecular signatures

**DOI:** 10.1038/s41586-021-03910-8

**Published:** 2021-10-06

**Authors:** Aparna Bhaduri, Carmen Sandoval-Espinosa, Marcos Otero-Garcia, Irene Oh, Raymund Yin, Ugomma C. Eze, Tomasz J. Nowakowski, Arnold R. Kriegstein

**Affiliations:** 1 Department of Neurology, University of California, San Francisco, San Francisco, CA USA; 2 The Eli and Edythe Broad Center of Regeneration Medicine and Stem Cell Research, University of California, San Francisco, San Francisco, CA USA; 3 Rebus Biosystems, Santa Clara, CA USA; 4 Department of Anatomy, University of California, San Francisco, San Francisco, CA USA; 5 Present Address: Department of Biological Chemistry, Los Angeles, Los Angeles, CA USA

**Keywords:** Developmental neurogenesis, Neural stem cells

## Abstract

The human brain is subdivided into distinct anatomical structures, including the neocortex, which in turn encompasses dozens of distinct specialized cortical areas. Early morphogenetic gradients are known to establish early brain regions and cortical areas, but how early patterns result in finer and more discrete spatial differences remains poorly understood^1^. Here we use single-cell RNA sequencing to profile ten major brain structures and six neocortical areas during peak neurogenesis and early gliogenesis. Within the neocortex, we find that early in the second trimester, a large number of genes are differentially expressed across distinct cortical areas in all cell types, including radial glia, the neural progenitors of the cortex. However, the abundance of areal transcriptomic signatures increases as radial glia differentiate into intermediate progenitor cells and ultimately give rise to excitatory neurons. Using an automated, multiplexed single-molecule fluorescent in situ hybridization approach, we find that laminar gene-expression patterns are highly dynamic across cortical regions. Together, our data suggest that early cortical areal patterning is defined by strong, mutually exclusive frontal and occipital gene-expression signatures, with resulting gradients giving rise to the specification of areas between these two poles throughout successive developmental timepoints.

## Abstract (web summary)

RNA-sequencing analysis of the prenatal human brain at different stages of development shows that areal transcriptional signatures are dynamic and coexist with developmental and cell-type signatures.

## Main

Understanding when brain regions acquire their unique features and how this specification occurs has broad implications for the study of human brain evolution, including species-specific developmental differences that may have contributed to the expansion of cortical areas such as the prefrontal cortex (PFC)^2^. It is also crucial for unravelling the pathology of neurodevelopmental and neuropsychiatric disorders that often preferentially affect specific brain regions and/or neocortical areas^3,4^. Early patterning of the developing telencephalon is orchestrated by morphogenetic gradients of growth factors including bone morphogenetic proteins, Wnts, sonic hedgehog and, most prominent in the cortex, fibroblast growth factor^3,5^. However, the molecular patterns that arise as a result of these gradients are less well understood.

## Atlas of human brain development

**Fig. 1:**
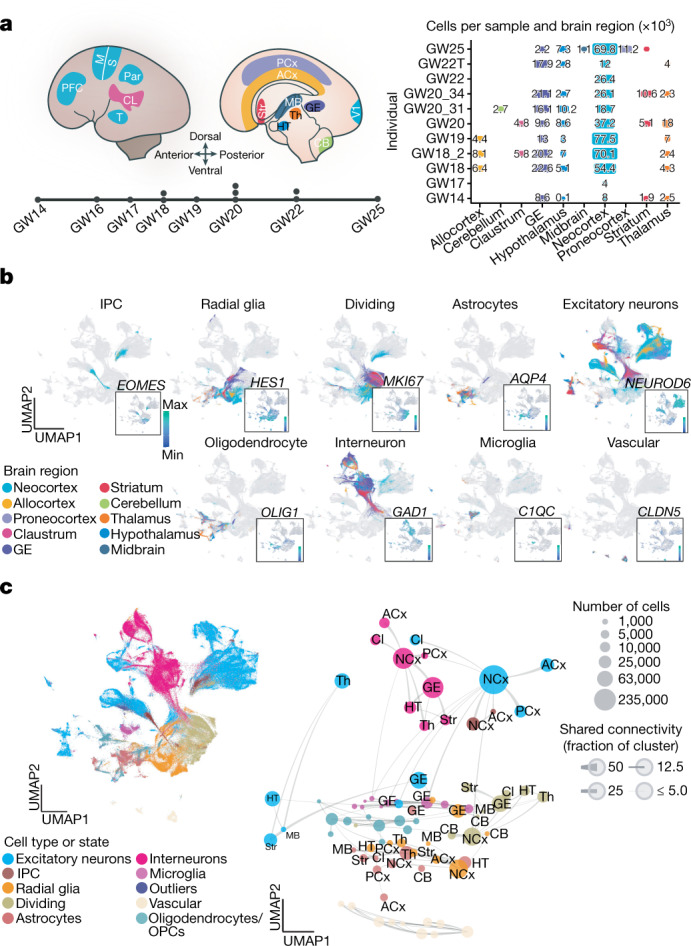
Single-cell analysis of gene-expression signatures across regions of the developing human brain. **a**, Left, schematic showing the anatomical brain regions sampled for this study. The timeline below highlights the number of individuals sampled at each gestational week. Right, matrix showing the final count (after quality control) of cells from each individual distributed across regions sampled. **b**, Single cells from all brain regions sampled are represented in UMAP space. Cells are colour-coded by their region of origin. Insets show the expression profile of canonical genes representative of each identity. **c**, Top left, distribution of cell types and states in UMAP space. Constellation plot of cells grouped by type or state and brain region highlights the interplay between cell type (node colour) and regional identity (node label). Nodes are scaled proportionally to the number of cells in each group. Edge thickness at each end represents the fraction of cells within a group with neighbours in the opposite group. Node colour corresponds to cell type or state; node label corresponds to the brain region from which cells were sampled. ACx, allocortex; CB, cerebellum; CL, claustrum; GE, ganglionic eminences; HT, hypothalamus; M, motor cortex; MB, midbrain; NCx, neocortex; Par, parietal cortex; PCx, proneocortex; S, somatosensory cortex; Str, striatum; T, temporal lobe; Th, thalamus.

**Extended Data Fig. 1:**
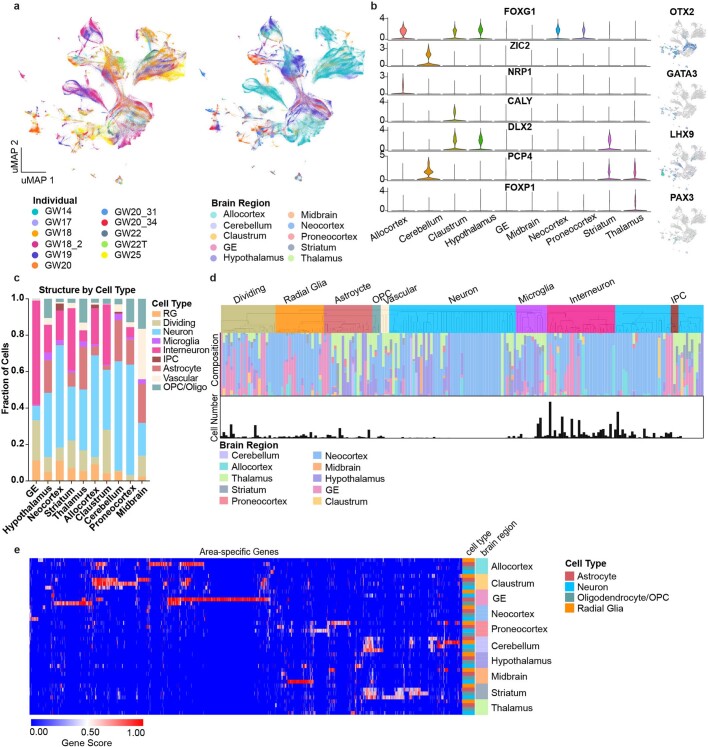
Single-cell analysis across the whole developing human brain. **a**, UMAP plots showing the representation of samples by individual and brain region. There is strong intermixing across individuals, but more segregation by stage. **b**, On the left, violin plots of known and novel brain region-enriched marker genes. On the right are feature plots of cell type specific transcription factors. **c**, Histogram depicting the cell type composition as determined by the single-cell analysis for each sampled brain region, showing similar distributions across regions, but with known enrichments for inhibitory interneurons in the ganglionic eminences, and other small enrichments for specific cell types in other regions. **d**, Hierarchical clustering of 210 neocortex clusters based upon Pearson correlations across cluster markers. Each bar is colored based upon the major cell type assigned to that cluster. Beneath the clusters are histograms showing the fraction of cells from each area contributing to the cluster, and below that is a barchart showing the relative number of cells in each cluster (log2 transformed numbers ranging from 0 to 20). **e**, Heatmap representing the universe of area-specific genes for each cell type. Gene score, a metric that combines specificity and fold change, is shown from blue to red. Rows are grouped by brain region, and reveal that in many structures, area-specific genes cross cell types.

To characterize the emergence of cellular diversity across major regions of the developing human brain and across cortical areas, we sequenced single-cell transcriptomes from microdissected regions of developing human brain tissue during the second trimester, which encompasses peak stages of neurogenesis^6^. We sampled cells from 10 distinct major forebrain, midbrain and hindbrain regions from 13 individuals (Fig. 1a, Supplementary Table 1, Methods). In addition, we sampled six neocortical areas from the same individuals: PFC, motor, somatosensory, parietal, temporal and primary visual (V1) cortex, resulting in 698,820 high-quality cells for downstream analysis. Here we refer to the subdivisions of the cerebrum and cerebellum as ‘regions’, and to subdivisions of the cerebral cortex as ‘areas’. Microdissections were performed carefully to sample target regions. However, it should be noted that these regions are putative during development, and small numbers of cells from neighbouring regions may have been included. We found expected cell populations including excitatory neurons, intermediate progenitor cells (IPCs), radial glia, mitotic cells, astrocytes, oligodendrocytes, inhibitory neurons, microglia and vascular cells (including endothelial cells and pericytes) (Fig. 1b, Extended Data Fig. 1a).

We found genes that were region-specific across all cell types, as well as genes that were region-specific for individual cell types (Supplementary Tables 2, 3). We detected previously described markers of brain regions, including *FOXG1* (cortex)^7^, *ZIC2* (cerebellum, also observed in the neocortex)^8,9^ and *NRP1* (allocortex)^10^ (Extended Data Fig. 1b, Supplementary Table 2). We also identified numerous cell type and structure-specific transcription factors, including *OTX2*, *GATA3*, *LHX9* and *PAX3*. Each region contained progenitor and differentiated cell types (Extended Data Fig. 1c), leading us to ask whether brain region or cell type is a stronger component of regional identity during the second trimester. At earlier developmental timepoints, we and others have noted that regional signatures are not broadly pervasive and do not yet reflect area-specific identities of unique brain substructures^11,12^. As expected, cluster branches were primarily organized by cell type, validating our annotation approach and highlighting the robustness of cell type in driving cluster similarity. However, quantifying the proportion of cells from each region contributing to each cluster showed that the majority (115 out of 192) of clusters were strongly enriched for a single or related brain region (Extended Data Fig. 1d).

We found that across the whole brain, cell type was the primary source of segregation, as visualized in constellation plots^13^ (Fig. 1c). However, in certain cases, such as the ganglionic eminence, cells of distinct types from a common region are drawn together in uniform manifold approximation and projection (UMAP) space, suggesting that regional identity can also be a strong source of variation (Fig. 1c). A heat map of area-specific gene score enrichments (Methods) shows that some region-specific genes are present across multiple cell types within a given region. This suggests that some regional gene-expression signatures are highly penetrant across cell types. Of note, we found that regionalization is stronger in glial populations (Extended Data Fig. 1e, Supplementary Table 3).

**Extended Data Fig. 2:**
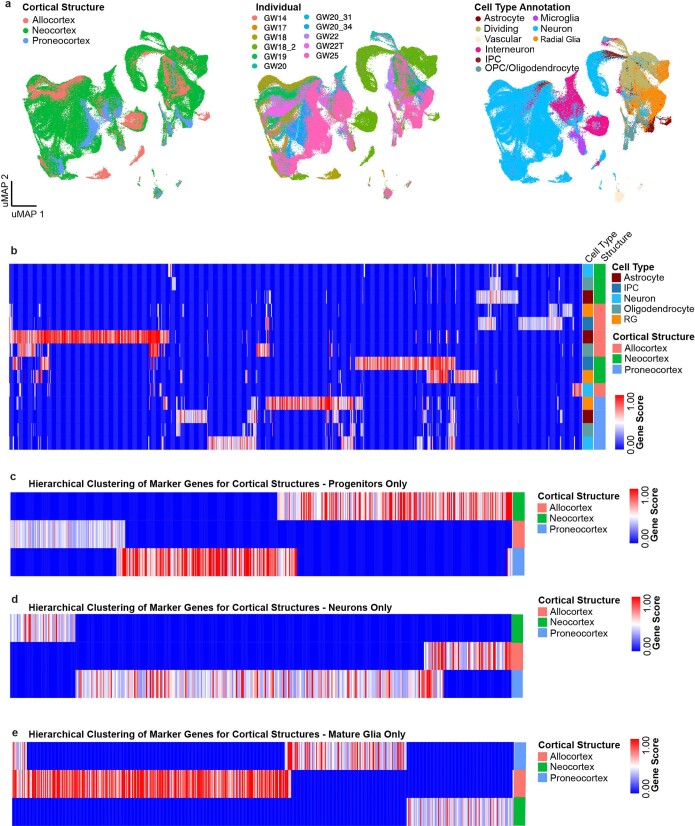
Single-cell analysis of regional identities across distinct cortical structures. **a**, UMAP projection of neocortex, allocortex and proneocortex cells alone, using cell type annotations from the previous analysis. Left UMAP plot shows integration of neocortex and allocortex cells (green and salmon, respectively), but exclusion of proneocortex cells (blue). In the middle UMAP plot, cells are colored by individual. Right UMAP plot shows the distribution and strong segregation by cell type. **b**, Marker genes (columns) specific to distinct cortical regions by cell type. Rows and columns are hierarchically clustered columns using one minus Pearson correlation for the distance metric. Heatmap reflects strong regional transcriptional identities, even among these 3 cortical structures. Additionally, it highlights expression signatures that cross cell type boundaries. **c**, Marker genes (columns) specific to distinct cortical regions analyzed in progenitors only. Rows and columns are hierarchically clustered columns using one minus Pearson correlation for the distance metric. **d**, Marker genes (columns) specific to distinct cortical regions analyzed in excitatory neurons only. Rows and columns are hierarchically clustered columns using one minus Pearson correlation for the distance metric. **e**, Marker genes (columns) specific to distinct cortical regions analyzed in mature glia only. Rows and columns are hierarchically clustered columns using one minus Pearson correlation for the distance metric.

The neocortex, allocortex and proneocortex are evolutionarily closely related and physically proximal^14^. We sought to identify distinct regional gene-expression programs among these three closely related regions by co-clustering these samples independently (Extended Data Fig. 2a). Surprisingly, even within these closely related cortical structures, region was still the primary driving force, and again, regional signatures bridged multiple cell types (Supplementary Table 4, Extended Data Fig. 2b–e). These analyses indicate that regional signatures are sufficiently established during the second trimester to distinguish cells across brain structures, with some signatures extending beyond an individual cell type.

## Cell types in the neocortex

**Fig. 2:**
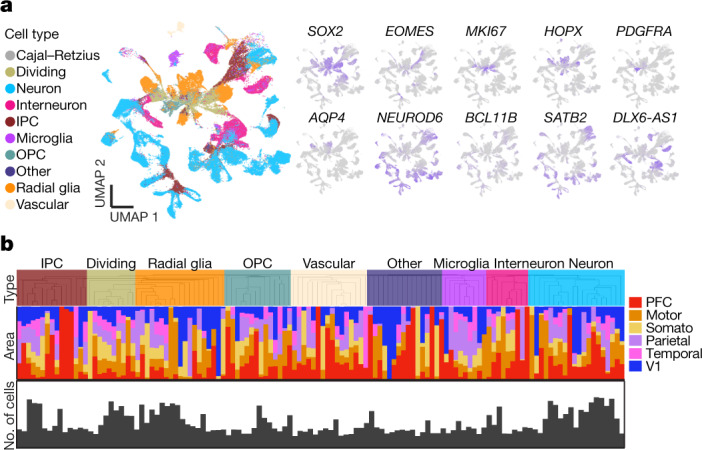
Cell types in the developing human neocortex across cortical areas. **a**, Single cells from the neocortex represented in UMAP space. Cells in the far left UMAP plot are colour-coded by cell type or state annotation. Feature plots on the right depict the expression pattern of major cell population markers (*SOX2*, radial glia; *EOMES*, IPC; *MKI67*, dividing cells; *HOPX*, outer radial glia; *PDGFRA*, OPC/oligodendrocyte; *AQP4*, astrocytes; *BCL11B*, deep layer excitatory neurons; *SATB2*, superficial layer excitatory neurons; *NEUROD6*, broad excitatory neurons; *DLX6-AS1*, inhibitory neurons). **b**, Hierarchical clustering of 138 neocortical clusters on the basis of the Pearson correlations of cluster marker expression profiles across all neocortical stages sampled. Branches are colour-coded by the major cell type assigned to each cluster group. Histograms below show the fraction of cells from each area contributing to a cluster. Bottom bar chart shows the relative number of cells (log_2_-transformed, range 0 to 20) in each cluster.

**Extended Data Fig. 3:**
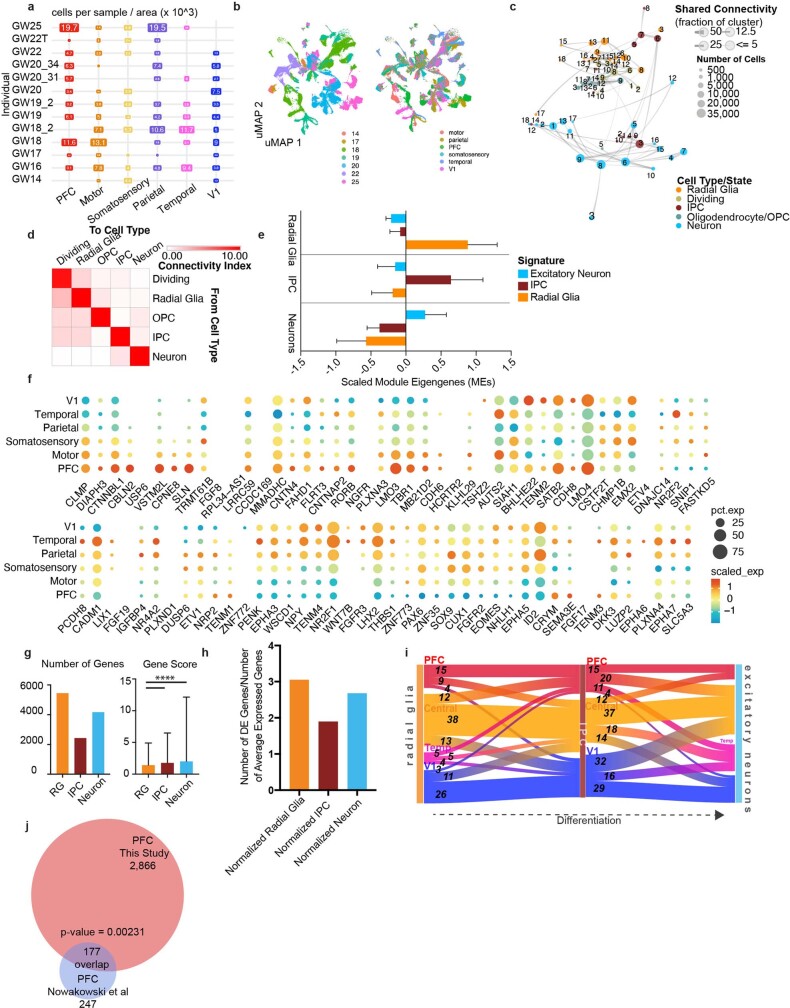
Single-cell analysis across the developing human neocortex. **a**, Matrix showing the distribution of the number of cells across areal dissections for each of the individuals samples. Number of cells is shown in boxes sized proportionally to the number of cells from that individual and region, times 1000. **b**, UMAP plots showing the representation of samples by age (GW) (left) and brain region (right). There is strong intermixing across individuals, but more segregation by stage. **c**, Constellation plot shows the relationships between clusters of the excitatory lineage and oligodendrocytes, highlighting the strong relationships between clusters of the same cell type and lineage, but not between those of different groups. Nodes are scaled proportionally to the number of cells in each group. Edge thickness at each end represents the fraction of cells within a group with neighbors in the opposing group. Nodes are colored by cell type/state, and labelled by the brain region from which cells were sampled. **d**, Constellation plot quantification, with ‘towards cell type’ connectivity as columns and ‘from cell type’ connectivity as rows. The connectivity index integrates the number of connections between two distinct cell types, as well as the average fraction of contributing cells from each cluster. **e**, Differential expression was performed at the cell type level between radial glia, IPCs, and excitatory neurons. Each set of cell-type-enriched genes was used to create a network and scaled module eigengenes were calculated for each cell type. n = 50,000 cells subsampled from full dataset. Mean with standard deviation error bars are shown. **f**, Dot plot of area-specific genes as identified in Cadwell, et al 2019 as they are expressed in our dataset. Most genes show expected expression patterns, though some deviate from these expectations, likely because many of these genes have been characterized in the rodent. **g**, Quantification of the number of differentially expressed areal genes from each cell type, using a union of all genes as calculated across each individual in the dataset. The gene score, an integration of fold change and specificity is quantified in the lower graph, with mean plus standard deviation shown. Neurons vs radial glia p-value = 0.000011; IPC vs radial glia p-value = 0.000435 (one-sided t-test). For both analyses, n= 5446 (radial glia), 2426 (IPCs), 4170 (Neurons). **h**, Quantification of the number of differentially expressed areal genes from each cell type, using a union of all genes as calculated across each individual in the dataset normalized by the average number of genes expressed within that cell type. **i**, Sankey plots show the proportion of area-specific gene groups shared between cell types and cortical areas. The number on each block line indicates how many genes are represented by that stream as the stream sizes are not to scale. The “central” area encompasses motor, parietal, and somatosensory areas. **j**, Venn diagram showing the overlap between the PFC/V1 genes in the Nowakowski, et al 2017 dataset and this study. p-value = 0.00231, Chi-square test. Source data

The neocortex comprises dozens of functional areas that specialize in wide range of cognitive processes^15^. Longstanding, juxtaposed hypotheses propose the existence of either a cortical protomap^16^, where the areal identity of cortical progenitors is cell-intrinsic and genetically predetermined, or a protocortex, where newborn neurons are not areally specified until extrinsic signals such as those from thalamocortical afferents reach the developing cortex^17^. Recent work has shown that while neurons are distinct between V1 and PFC soon after their birth^18^, other cell types do not show robust area-specific differences. Studies in the adult mouse have additionally shown that while neuronal cell types of the anterior lateral motor cortex (ALM) and V1 are transcriptionally distinct from each other^1^, denser sampling of areas between the ALM and V1 reveals a gradient-like transition between cell-type profiles^19^. We sought to expand upon these findings by profiling single cells from distinct cortical areas, yielding 387,141 high-quality cells, after filtering (Methods) (Extended Data Fig. 3a, b). We found expected cell types, including Cajal–Retzius neurons, dividing cells (expressing division programs in addition to other cell type identities), excitatory neurons, inhibitory neurons, IPCs, microglia, oligodendrocyte precursor cells, radial glia/astrocytes and vascular cells (Fig. 2a, Supplementary Tables 5, 6). Hierarchical clustering of 138 neocortical clusters grouped cells by cell type (Fig. 2b) and revealed that most clusters (104 out of 138) are composed of cells from multiple cortical areas.

We found strong transcriptional proximity between clusters of the same cell type, suggesting that borders between clusters are fluid (Extended Data Fig. 3c). To quantify intra-cell type and inter-cell type similarity, we calculated the magnitude of transcriptional proximity between nodes (Methods), and found, not surprisingly, that clusters of each cell type connected most strongly to each other (Extended Data Fig. 3d). However, we also found that IPC subclusters connected much more strongly with excitatory neurons than with radial glia subclusters (Extended Data Fig. 3d). We then defined gene signatures characteristic of radial glia, IPCs and excitatory neurons using a differential gene-expression approach (Methods). These signatures (Supplementary Table 7) were scored using a module-eigengene calculation (Methods). We found that radial glia had the highest up-regulation of the progenitor signature, but lower down-regulation of the IPC and neuronal signatures (Extended Data Fig. 3e).

## Dynamic areal signatures

**Fig. 3:**
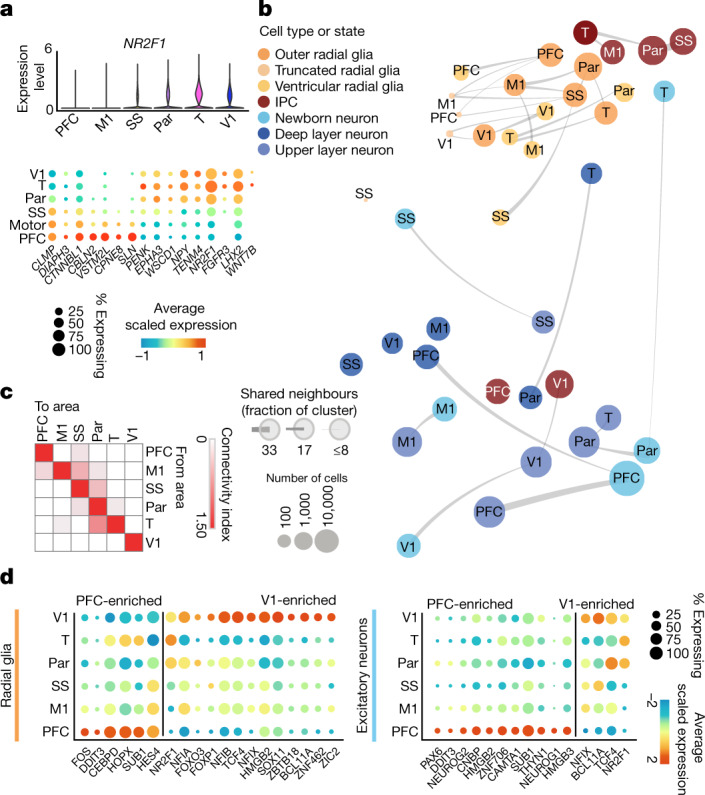
Cortical area-specific gene signatures. **a**, Top, violin plots show the expression of the previously described posterior-high to anterior-low gradient marker gene *NR2F1* across all neocortical cells grouped by area. Bottom, dot plot shows the expression of a representative panel of previously reported areally enriched genes across all neocortical cells grouped by area. Expression profiles validate the areal identity of the cortical subdissections used in this study. **b**, Constellation plots of excitatory lineage grouped by cortical area and annotated by cell subtype highlight cascading differences in areal identity, with similarities between cell types from the same region. Each dot is scaled proportionally to the number of cells represented by that analysis. The thickness of the connecting line on each end represents the fraction of cells within each group with neighbours in connected groups. Dot colour represents cell type and text over the dot marks cortical area. **c**, Quantification of the constellation plots, with ‘towards area’ in columns and ‘from area’ in rows. The connectivity index from white to red integrates the number of connections between two cell types as well as the average fraction of cells from each cluster contributing to each connection. **d**, Dot plots quantify a subset of transcription factors enriched in PFC or V1 radial glia (left) and excitatory neurons (right) relative to other cortical areas. Enrichment can occur through an increase in the number of cells expressing a given gene, an increase in the average expression level of expressing cells, or both. Cortical areas: M1, motor; Par, parietal; SS, somatosensory; T, temporal; V1, visual.

To explore areal differences amongst cells in the developing neocortex, we looked for differentially expressed genes for each cell type in the excitatory lineage (radial glial (RG), IPCs and excitatory neurons) across cortical areas. We validated cortical area sub-dissections by quantifying the expression of *NR2F1*, which has a posterior-high to anterior-low expression gradient in neocortex^19^, as well as other previously described area-specific genes (Fig. 3a, Extended Data Fig. 3f).

**Extended Data Fig. 4:**
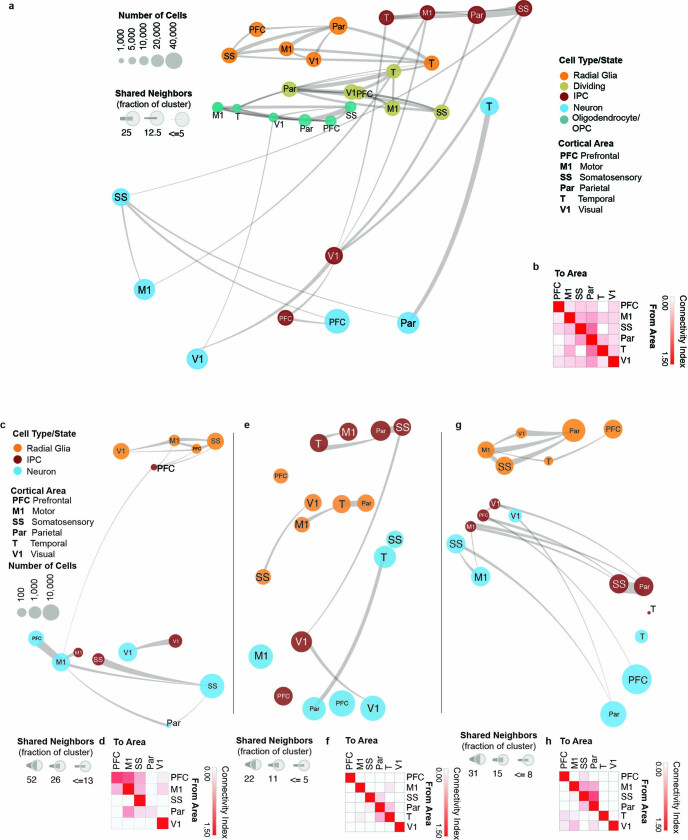
Constellation plots cell types and developmental stages. **a**, Constellation plots of excitatory lineage and oligodendrocyte lineage cell grouped by type and cortical area highlight similarities between groups of frontal and occipital cortical areas. Each dot is scaled proportionally to the number of cells represented by that analysis. The thickness of the connecting line on each end represents the fraction of cells within each group with neighbors in connected groups. Dot color represents cell type while text over the dot marks cortical area. **b**, Quantification of the constellation plots, with ‘towards area’ in columns and ‘from area’ in rows. The connectivity index from white to red integrates the number of connections between two cell types as well as the average fraction of cells from each cluster contributing to each connection. **c**, Constellation plots of excitatory lineage grouped by cortical area within early (GW14 – GW17) samples. **d**, Quantification of the early constellation plots. **e**, Constellation plots of excitatory lineage grouped by cortical area within mid-stage (GW18 – GW20) samples. **f**, Quantification of the mid-stage constellation plots. **g**, Constellation plots of excitatory lineage grouped by cortical area within late-stage (GW18 – GW20) samples. **h**, Quantification of the late-stage constellation plots.

Consistent with prior observations^17^, the specificity of neuronal areal markers was significantly higher compared with RG (Extended Data Fig. 3g). Surprisingly, however, more genes were differentially expressed in RG across areas than in neurons (Extended Data Fig. 3h, i, Supplementary Table 8). In addition to novel area-specific genes, our dataset also contains area-specific genes that overlap with those found in previous studies (Extended Data Fig. 3j). To explore the relationships between cell types from distinct cortical areas, we built constellation plots between nodes corresponding to each area and cell type combination. RG nodes were connected predominantly to each other, whereas IPCs and excitatory neurons were frequently interconnected, especially within the same neocortical area (Fig. 3b, c). Of note, neuronal nodes of different areas show robust area-specific transcriptional proximity to their IPC counterparts, suggesting that some degree of the areal specification seen in neurons may be already present in IPCs (Extended Data Fig. 4a, b). We did not find edges between PFC and V1 cell-type nodes (Fig. 3c, Extended Data Fig. 4b), pointing to a model of strong mutual exclusivity between these two gene-expression programs. Cell subtypes also followed this pattern; PFC and V1 outer radial glia cells are already mutually exclusive, and newborn neurons connect primarily to more mature neurons from the same area. These patterns persist across broader cell type annotations (RG and excitatory neurons), as well as across individual developmental stages (early, middle and late second trimester) (Extended Data Fig. 4a–h). These observations indicate that markers of areal identity are already detectable in RG but become more pronounced as differentiation proceeds: we found that area-specific gene-expression signatures change substantially across cell types, with small numbers of areal markers preserved throughout differentiation (Extended Data Fig. 3i).

**Extended Data Fig. 5:**
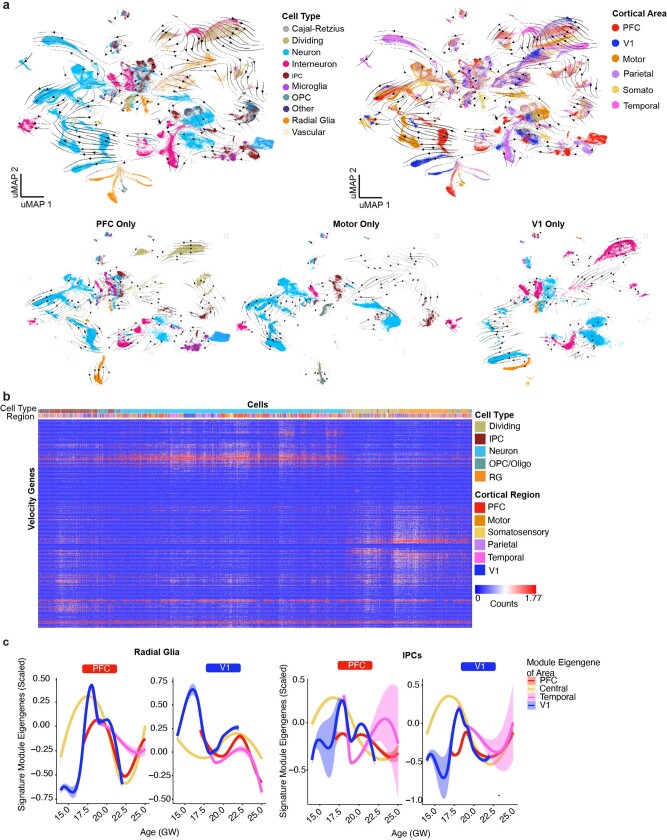
Trajectories of differentiation and changes in gene signatures. **a**, RNA velocity analysis was performed using all necortical cells from the dataset. Stream arrows depict predicted differentiation trajectories. Cells are shown in UMAP space, and are colored by cell type (left panel) and by cortical area (right panel). In the second row, zoomed in streams are shown for individual cortical areas PFC, motor and V1 only colored by cell type. **b**, Normalized counts for area-specific genes (rows) that were detected by the RNA-velocity algorithm are shown across a random subset of 20,000 cells (columns). Rows and columns were hierarchically clustered using a one minus pearson correlation distance matrix. Cell type and region are shown for each cell (top color bar), and highlight cortical area specific excitatory neuron populations. **c**, Module eigengenes were calculated for radial glia (left) and IPCs (right) based upon the area specific gene signatures identified in neurons of that area. For both sets of plots, the gene-expression signature of PFC neurons is shown on the left and the V1 signature is shown on the right. Lines indicate the signature strength for progenitors of each region across the timepoints sampled. Early in development, PFC radial glia are defined by a low V1 signature strength, while V1 radial glia are defined by a strong V1 signature and minimal signal for all other areal signatures. IPCs from the PFC and V1 are generally defined by the lack of a strong signature for any of the areas calculated. Range shows 95% confidence interval.

To further investigate the relationship between cell differentiation and areal signature dynamics, we inferred lineage trajectories using RNA velocity^20,21^ (Extended Data Fig. 5a). For each cortical area, we identified the most dynamic genes across the differentiation cascade as the top-loading RNA velocity genes (Extended Data Fig. 5b). We found a large enrichment of genes in excitatory neurons, but not in RG or IPC populations, leading us to question how areal signatures might change as cells differentiate. We defined areal signatures of excitatory neurons as gene networks and evaluated their strength in RG across cortical areas by calculating their module eigengene scores. We found a strong early binary V1 expression, while the PFC signature emerged only later (Extended Data Fig. 5c).

**Extended Data Fig. 6:**
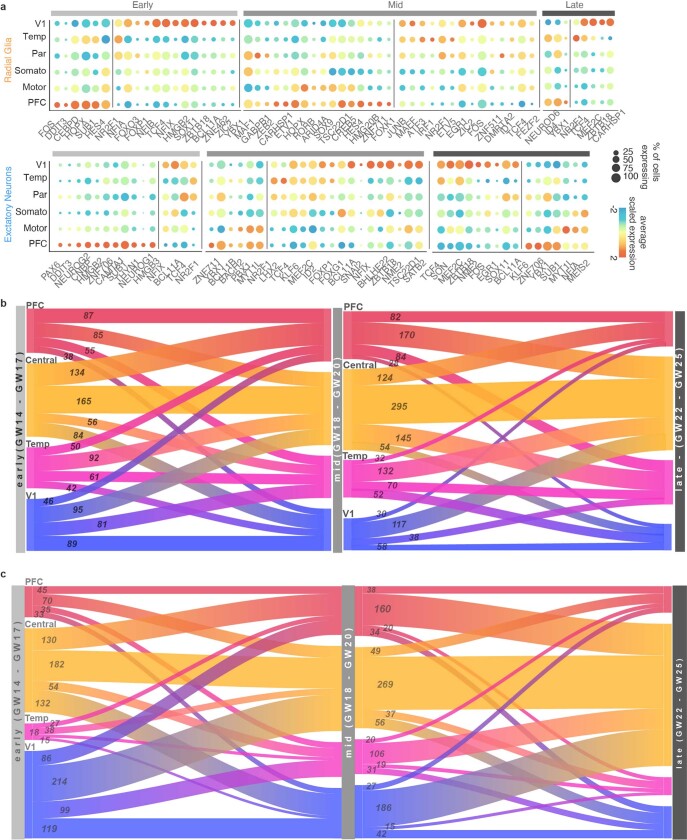
Gene-Expression Dynamics Across Areas and Developmental Stages. **a**, Dot plots transcription factors enriched across areas in radial glia (left) and excitatory neurons (right) relative to other cortical areas. Enrichment can occur via an increase in the number of cells expressing a given gene, an increase in the average expression level of expressing cells, or both. **b**, Sankey plots show the proportion of area-specific excitatory neuron genes shared across developmental stages. The number on each block line indicates how many genes are represented by that stream as the stream sizes are not to scale. The “central” area encompasses motor, parietal, and somatosensory areas. **c**, Sankey plots show the proportion of area-specific radial glia genes shared across developmental stages. The number on each block line indicates how many genes are represented by that stream as the stream sizes are not to scale.

Within each set of areal marker genes, we identified genes encoding transcription factors that were robustly enriched in cells of a specific area as well as transcription factors with a broad frontal or caudal enrichment (Fig. 3d). A subset of area-specific transcription factors showed consistent specificity through early, middle, and late second trimester (Extended Data Fig. 6a). We detected genes for transcription factors with known roles in arealization, such as *NR2F1*, which confers positional identity across the rostro-caudal axis^19^, and the gene encoding BCL11A, which interacts with NR2F1 and represses motor cortex identity^22^. Both genes are implicated in neurodevelopmental disease^23,24^. Additionally, we detected genes encoding transcription factors that have not been implicated in cortical arealization. In V1, these include *NFIA*, *NFIB* and *NFIX*, which are important regulators of brain development implicated in macrocephaly and severe cognitive impairment^25^. They also include *ZBTB18* (also known as *RP58*), a putative driver of brain expansion involved in neuron differentiation and cortical migration^26,27^. In the PFC, area-specific transcription factors include HMGB2 and HMGB3, which are differentially expressed by neural stem cells at distinct stages of development^28^ and are thought to be key regulators of differentiation. Of note, HMGB3 mutations can result in severe microcephaly. We also found upregulation of the genes encoding the transcription factors *NEUROG1* and *NEUROG2* in PFC neurons. Although these PFC-specific genes have been previously described as regulators of neuronal differentiation, they have not been implicated or studied in the process of cortical arealization.

**Extended Data Fig. 7:**
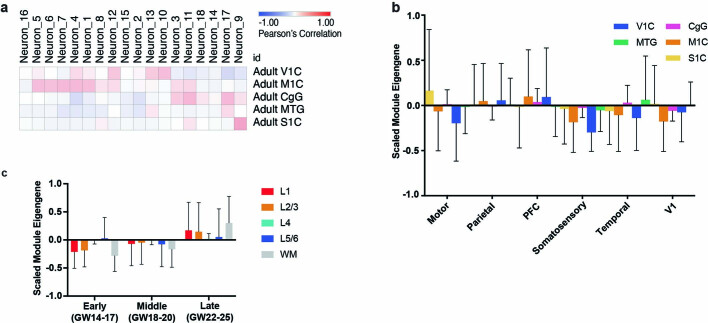
Signature Correspondence Between Developmental and Adult Samples. **a**, Correlations were performed between the neuronal clusters in this dataset and the area specific identities calculated from adult human cortical areas as obtained from the Allen Institute Brain Map dataset. **b**, Module eigengene calculations of adult areal signatures show minimal to no correspondence to the signatures we identify in this study based upon low module eigengene values. n = 50,000 cells subsampled from full dataset. Mean with standard deviation error bars are shown. **c**, Module eigengene values of Allen excitatory neurons layer specific signatures in each of the developmental stages within our dataset. n = 50,000 cells subsampled from full dataset. Upper layer signatures emerge at late stages, while a small signal for deep layer identities can be observed at the earliest stages. Mean with standard deviation error bars are shown. Source data

Consistent with proposed models of extensive transcriptional remodelling during the second and third trimesters^20^, we observed that while area-specific gene signatures are composed of significant and specific marker genes, they also change substantially throughout this period (Extended Data Fig. 6b, c). Concordantly, we only found a small overlap of area-specific gene signatures, and low cluster correspondence, between this dataset and that of the adult brain (Extended Data Fig. 7a–c). We thus find strong evidence for a partial early cortical protomap, which is then further refined as proposed by the protocortex model.

## In situ validation of neuronal markers

**Fig. 4:**
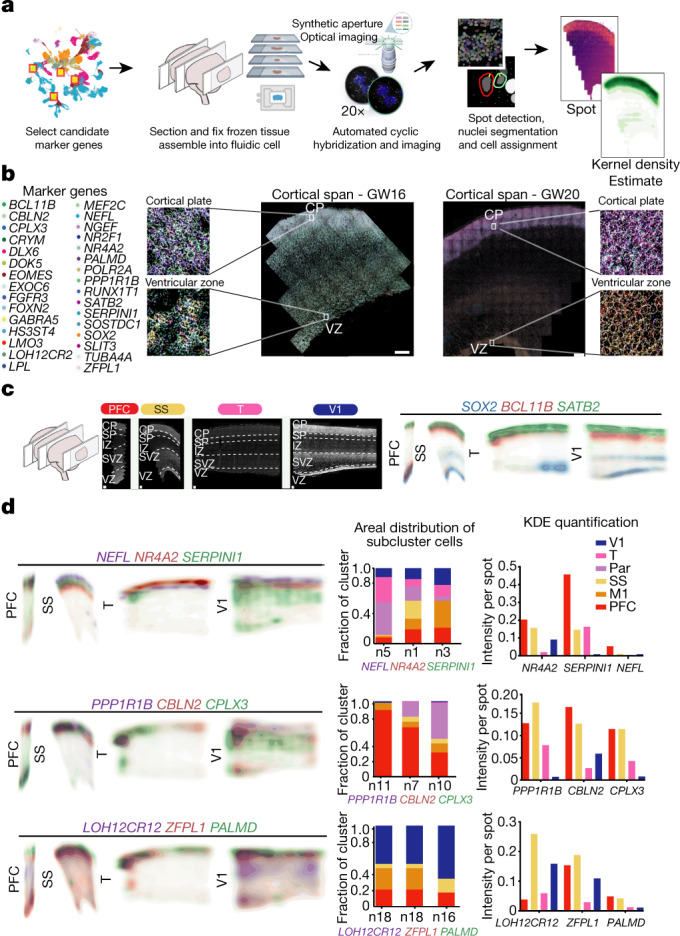
Spatial RNA analysis identifies distinct spatial patterns of area specific clusters. **a**, Automated spatial RNA transcriptomics workflow used to validate the expression patterns of candidate marker genes in situ across four distinct cortical areas. Tissue blocks from 4 cortical areas of a GW20 and a GW16 sample were sectioned (7–10 µm in thickness) onto coverslips and mounted into a fluidic chamber, in which iterative smFISH was performed in batches of 3 genes at a time. RNA molecules were quantified and assigned to individual cells by automated spot detection and nuclei segmentation. **b**, Representative merged images of smFISH for 31 candidate marker genes in a GW16 (left) and GW20 (right) somatosensory cortex section. Zoomed in images of the ventricular zone (left) and cortical plate (right). White circles indicate segmented nuclei. This analysis was performed once for each of the four regions. Scale bar, 444 μm. **c**, Top left, nucleus staining outlines tissue architecture, with the ventricular zone at the bottom and the cortical plate at the top. Top right, KDE plots for positive-control genes. CP, cortical plate; IZ, intermediate zone; SP, subplate; SVZ, subventricular zone; VZ, ventricular zone. Scale bar, 444 μm. **d**, KDE plots for neuronal genes of interest. Genes were chosen as candidate markers for specific neuronal subclusters. Clusters being explored are named below the histogram and the gene marker for the cluster is shown below its name. Stacked histograms show the expected ratio of clusters as a fraction of total composition. Right, KDE plots are quantified as intensity divided by the number of spots to reflect both the intensity of signal and the pervasiveness of the marker to not artificially bias the analysis owing to rare but intense signals. Source data

**Extended Data Fig. 8:**
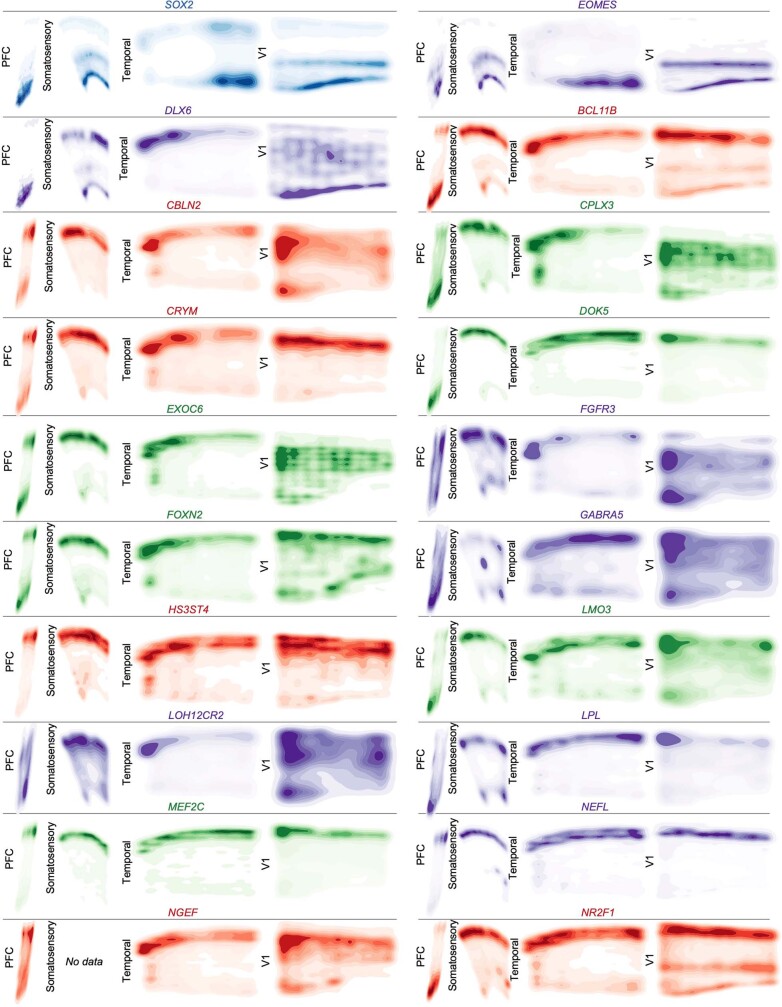
Spatial Expression Patterns of Cell Type and Neuronal Cluster Marker Genes GW20 Part 1. Kernel density plots of each gene assayed using spatial RNA *in situ* analysis. Plots are made from all spots, and are shown across all four sampled cortical areas of a GW20 individual. Color is for emphasis of expression, but individual colors have no specific meaning.

**Extended Data Fig. 9:**
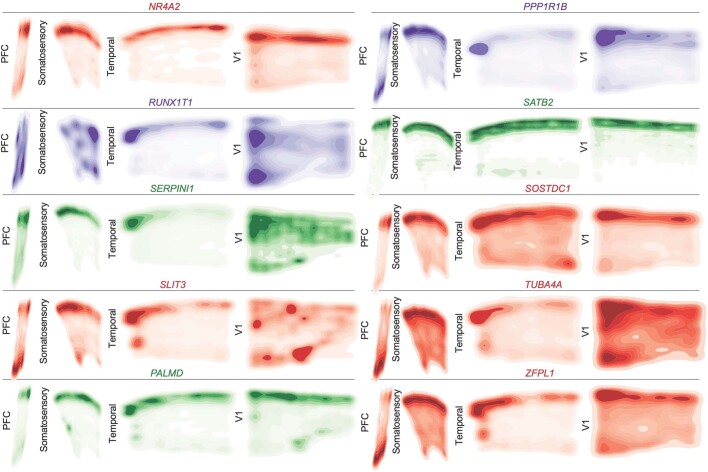
Spatial Expression Patterns of Cell Type and Neuronal Cluster Marker Genes GW20 Part 2. Kernel density plots of each gene assayed using spatial RNA *in situ* analysis. Plots are made from all spots, and are shown across all four sampled cortical areas of a GW20 individual. Color is for emphasis of expression, but individual colors have no specific meaning.

Our single-cell data uncover a large diversity of cell types and transcriptional profiles across six areas of the developing human cortex. We selected candidate markers of excitatory neuron clusters that were enriched in one or more sampled areas for validation by multiplexed single-molecule fluorescent in situ hybridization (smFISH) (Fig. 4a). We quantified the expression level of 31 RNA transcripts per tissue section in four cortical regions from a gestation week (GW)20 sample (Fig. 4b). We used DAPI staining along with kernel density expression (KDE) plots^21^ of canonical cell type marker genes (*SOX2*, *SATB2* and *BCL11B*) to identify the ventricular zone and cortical plate (Fig. 4c, Extended Data Fig. 8, 9). We confirmed previously described areal pattern dynamics between the neuronal genes *SATB2* and *BCL11B*, which are co-expressed in frontal regions but mutually exclusive in occipital areas^18^ (Fig. 4c). These spatial datasets are available at https://kriegsteinlab.ucsf.edu/datasets/arealization (Supplementary Tables 9–12).

**Extended Data Fig. 10:**
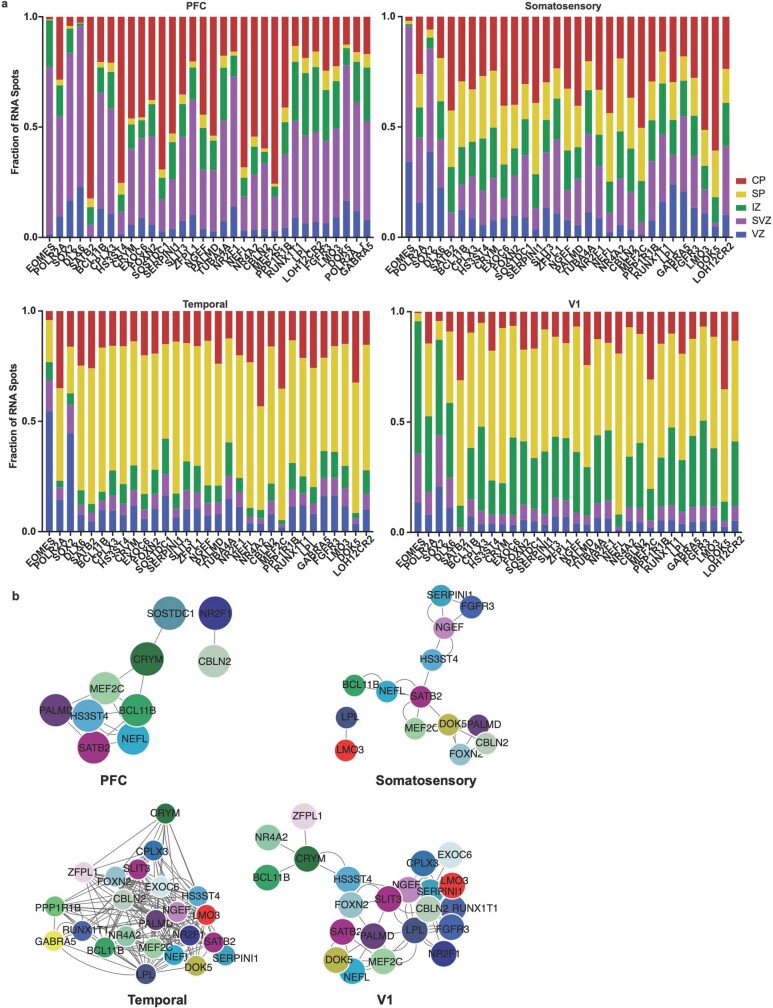
Laminar and network changes across cortical areas (GW20). **a**) We quantified the laminar distributions of each gene in each GW20 sample. The distributions are shown by laminar region, as annotated in Fig. 4, and are represented as fraction of signal for each gene in each bin. **b**) Using cell identities for individual gene spots, we computed co-expression networks for each sample to visualize changes in the co-expression patterns of the 31 genes analyzed across areas of the cortex. Gene pairs were classified as coexpressed if their Pearson correlation was >=0.05. Networks are shown in a force-directed layout reflective of interaction strength. Only neuronal marker genes are shown, colored as in panel (b).

Across all areas, we explored novel candidate subpopulation markers, including predicted subplate markers *NEFL, SERPINI1* and *NR4A2*. All three markers showed largely equal intensity levels across cells in PFC, somatosensory, temporal, and V1 cortex, but their relative spatial distribution changed substantially (Fig. 4d). These genes were co-expressed in PFC, but were mutually exclusive across all other regions. However, in the somatosensory cortex, these markers were expressed in upper cortical layers rather than in the subplate. Similarly, the spatial expression patterns of three frontally enriched marker genes, *PPP1R1B*, *CBLN2* and *CPLX3*, revealed higher signal in PFC and somatosensory cortex (Fig. 4d). Caudally, we observed higher intensities of *LOH12CR12*, *ZFPL1* and *PALMD* (Fig. 4d). We found marked differences in the laminar distribution of gene expression, suggesting that in addition to variable gene-expression levels across the rostro-caudal axis, laminar cell type distributions are also spatially dynamic (Extended Data Fig. 10a). While this observation may be reflective of differences in maturation states across the developing cortex, cell types may express genes in a different manner across distinct cortical areas.

**Extended Data Fig. 11:**
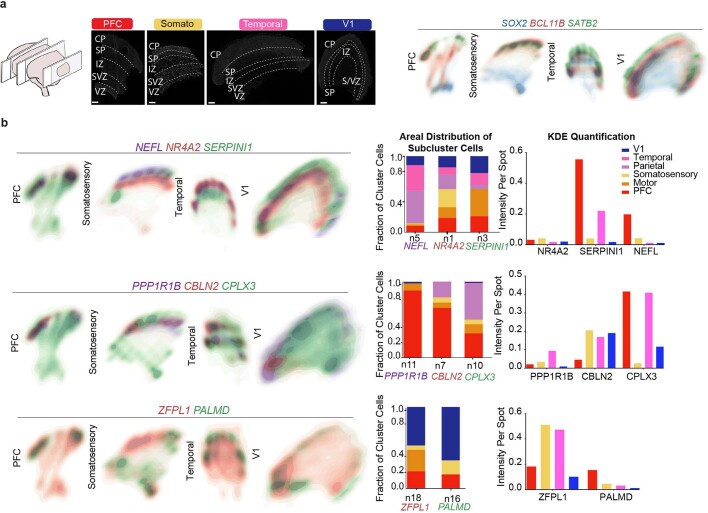
Spatial analysis of GW16 sample. **a**) Top left: Nucleus staining outlines tissue architecture, with the ventricular zone at the bottom and the cortical plate at the top. Top right: KDE plots of positive control genes. SOX2 marks radial glia and the ventricular zone, while SATB2 and BCL11B mark the cortical plate. As previously described, SATB2 and BCL11B are co-expressed in frontal regions, but are mutually exclusive in occipital regions. Scale bar = 444 μm. This analysis was performed once for each of the four regions. **b**) KDE plots of neuronal genes of interest. Genes were chosen as candidate markers for specific neuronal subclusters. Clusters being explored are named below the histogram each gene marker for this cluster is below its name. Stacked histograms show the expected ratio of clusters as a fraction of total composition. To the far right in each row, the quantification of the KDE plots is shown as intensity divided by the number of spots in order to reflect both the intensity of signal but also the pervasiveness of the marker to not artificially bias the analysis by examples of rare but intense signal. We see strong correspondence between the predicted spatial distribution of clusters and the signal in our spatial RNA analysis.

**Extended Data Fig. 12:**
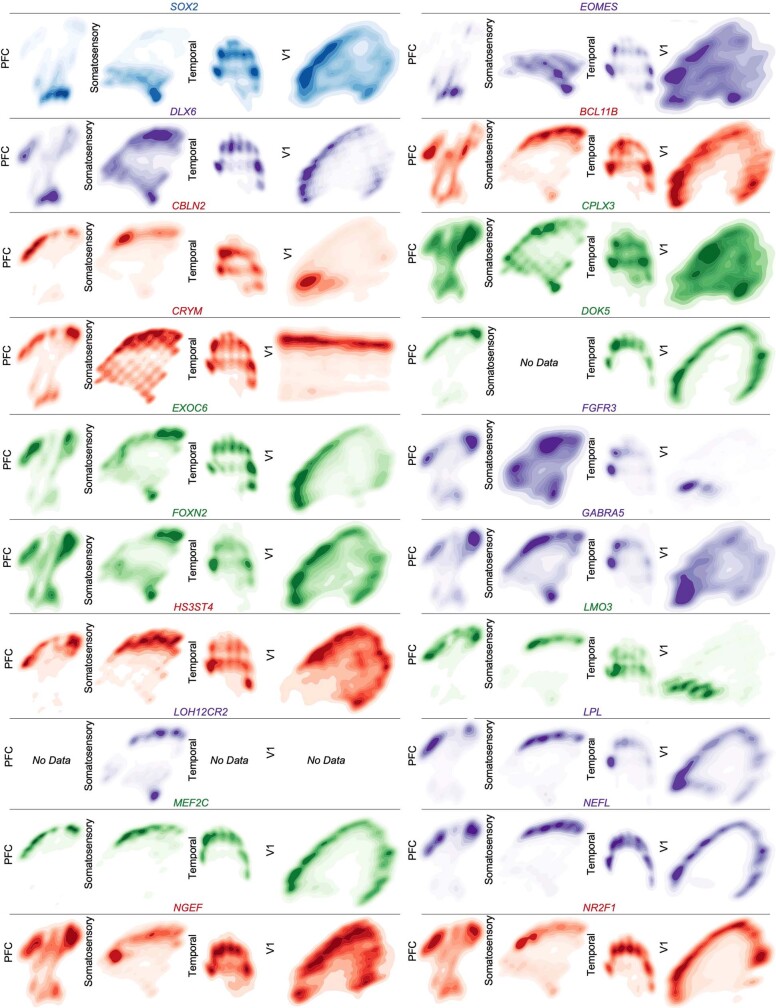
Spatial Expression Patterns of Cell Type and Neuronal Cluster Marker Genes GW16 Part 1. Kernel density plots of each gene assayed using spatial RNA *in situ* analysis. Plots are made from all spots and are shown across all four sampled cortical areas of a GW16 individual. Color is for emphasis of expression, but individual colors have no specific meaning.

**Extended Data Fig. 13:**
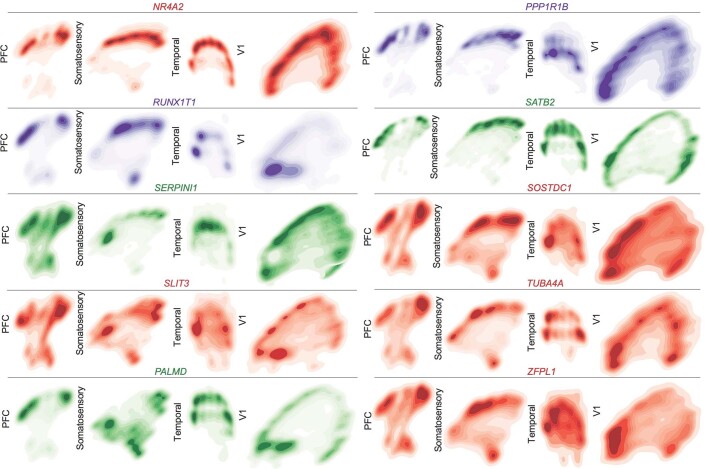
Spatial Expression Patterns of Cell Type and Neuronal Cluster Marker Genes GW16 Part 2. Kernel density plots of each gene assayed using spatial RNA *in situ* analysis. Plots are made from all spots, and are shown across all four sampled cortical areas of a GW16 individual. Color is for emphasis of expression, but individual colors have no specific meaning.

**Extended Data Fig. 14:**
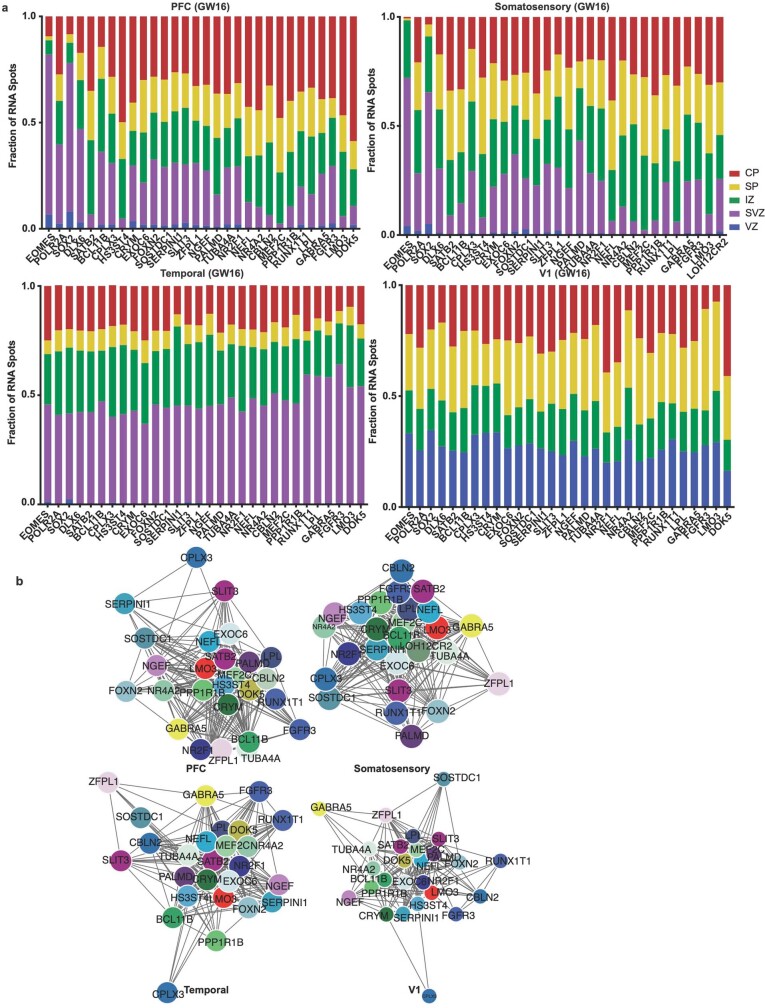
Laminar and network changes across cortical areas (GW16). **a**) We quantified the laminar distributions of each gene in each GW16 sample. The distributions are shown by laminar region, as annotated in Fig. 4, and are represented as fraction of signal for each gene in each bin. **b**) Using cell identities for individual gene spots, we computed co-expression networks for each sample to visualize changes in the co-expression patterns of the 31 genes analyzed across areas of the cortex. Gene pairs were classified as coexpressed if their Pearson correlation was >=0.05. Networks are shown in a force-directed layout reflective of interaction strength. Only neuronal marker genes are shown, colored as in panel (b).

We calculated co-expression relationships between single cells to generate networks that show the frequency of two genes expressed by the same cell (Extended Data Fig. 10b). The resulting networks highlight that the most stringent markers of areal identity are binary—that is, they are either included or excluded from the gene network. In most cases, however, we found remodelled co-expression patterns across cortical areas rather than elimination or inclusion of single genes from the network. Even when using all 31 genes to construct the networks, we see substantial co-expression remodelling across cortical areas. We replicated our spatial transcriptomics experimental workflow and analysis in a second individual (GW16), with the same results (Extended Data Figs. 11–14, Supplementary Tables 13–16).

## Discussion

**Extended Data Fig. 15:**
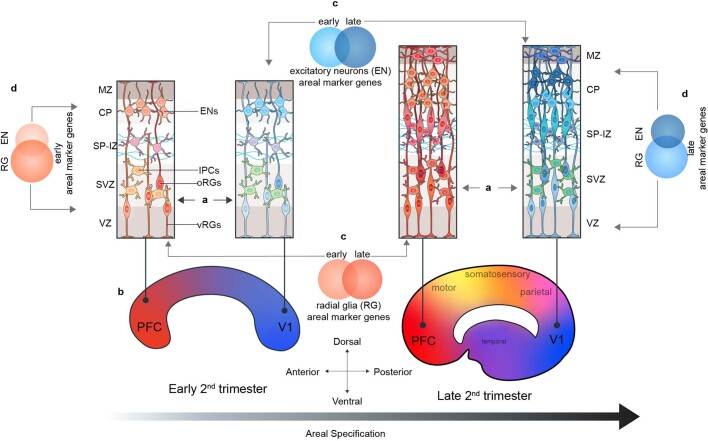
Summary Schematic of Proposed Model of Arealization. In this study, we find that in addition to their neuronal progeny, radial glia from distinct cortical areas are already distinct from each other (A). At early second trimester timepoints, the strongest contrast is seen radial glia at the frontal and occipital poles of the neocortex (B). The gene-expression signatures of cells at different cortical areas are highly dynamic across developmental time (C) and across the differentiation axis (RG → IPC → excitatory neuron) (D). As differentiation progresses, these dynamic gene signatures give rise to other major cortical areas, and further refinement occurs, likely as sensory input to the cortex begins to take place.

Our results provide a granular understanding of the gene-expression signatures of distinct cell types across neocortical areas throughout the second trimester of development. We find that across major brain structures, regional identity is highly pervasive among distinct cell types. By contrast, areal identity in the neocortex is highly specific and restricted to individual cell types. Furthermore, we find that in addition to cell-type identity, the developmental stage of cells (that is, gestational week) is a strong determinant of gene-expression signature composition. Together, these observations suggest that the dynamics of area-specific gene-expression signatures are surprisingly fast moving and cell-type-specific (Extended Data Fig. 15). This is in contrast to previous models of areal patterning, in which gene-expression programs have generally been assumed to be persistent once established.

We find strong evidence for the presence of a partial early cortical protomap between cell populations, including progenitors, at the frontal and occipital poles of the neocortex (Extended Data Fig. 15a, b). We see evidence of transcriptional regulation programs that may prime more differentiated and mature cells to acquire either a rostral or caudal identity. For example, even though progenitor clusters in the neocortex show little molecular diversity reflective of the multiple cortical regions that will eventually emerge, we do observe strong specification of PFC and V1 molecular identity among progenitor cells. In a previous study, we noted that radial glia were characterized by a small number of transcriptional differences that cascade into strong area-specific gene expression in excitatory neurons^18^. The analysis of a much larger number of cells and more cortical areas reveals a strong difference between PFC and V1 radial glia, while confirming that glutamatergic neurons are even more distinct between cortical areas. Our data suggest that cells located in between the prefrontal and occipital poles are less specified towards a particular areal identity, an observation that is more consistent with the protocortex hypothesis.

Characterizing the dynamic diversity of cell populations during the development of a structure as complex as the brain involves disentangling multiple axes of variation. Transcriptomic data can only provide hypotheses of how arealization occurs; mechanisms of actual specification cannot be tested without the use of model organisms and in vitro systems. This continues to present a challenge in the field because of the increased areal complexity of the human brain compared with rodent counterparts. The data we present here provides a spatially and temporally detailed molecular atlas of human brain and neocortex specification upon which future experimental characterizations can expand.

## Methods

### Sample acquisition

De-identified tissue samples were collected with previous consent in strict observance of the legal and institutional ethical regulations. Protocols were approved by the Human Gamete, Embryo, and Stem Cell Research Committee (institutional review board) at the University of California, San Francisco. Two sets of samples included twins: GW20_31 and GW20_34; GW22 and GW22T.

### Single-cell RNA sequencing capture and processing

Brain dissections were performed under a stereoscope with regards to major sulci to identify cortical regions. Of note, all dissections were performed by the same individual (T.J.N.) to enable reproducibility and comparison between samples. Tissue was incubated in 4 ml of papain/DNAse solution (Worthington) for 20 min at 37 °C, after which it was carefully triturated with a glass pipette, filtered through a 40-µm cell strainer and washed with HBSS. The GW22 and GW25 samples were additionally passed through an ovomucoid gradient (Worthington) in order to minimize myelin debris in the captures. The final single-cell suspension was loaded onto a droplet-based library prep platform Chromium (10X Genomics) according to the manufacturer’s instructions. Version 2 was used for all samples except for GW19_2, GW16, and GW18_2 for which version 3 chemistry was used. cDNA libraries were quantified using an Agilent 2100 Bioanalyzer and sequenced with an Illumina NovaSeq S4.

### Quality control and filtering

We filtered cells using highly stringent quality control (QC) metrics. In brief, we discarded potential doublets using the R package scrublet^29^ for each individual capture lane, then required at least 750 genes per cell and removed cells with high levels (>10%) of mitochondrial gene content. These strict metrics for quality control preserved no more than 40% of cells for downstream analysis, and re-analysis of the data for specific brain structures or cell types may benefit from less stringent QC for additional discovery. Our goal was to obtain clean populations with a high validation rate for a better understanding of arealization signatures. The resulting ~700,000 cells passing all thresholds were used in downstream analyses.

### Clustering strategy

We used a recursive clustering workflow to understand the cell types present in our dataset. In order to minimize potential batch effects and to increase detection sensitivity of potential rare cell populations, we performed Louvain–Jaccard clustering on each individual sample first. After initial cell type classification, we sub-clustered all the cells belonging to a cell type to generate the most granular cell subtypes possible. We then correlated subtypes between individuals based upon the gene scores in all marker genes to bridge any batch effects, and iteratively combined clusters across all individuals and cell types. For this study, we combined the clusters within a single cell type across all individuals once, and again with all clusters from all individuals and cell types, resulting in two iterative combinations. The annotations at each step are preserved in the supplementary tables to enable reconstruction at any point in the pipeline.

### Hierarchical clustering of clusters

Cluster hierarchies are generated from matrices correlating all clusters to one another using Pearson’s correlation in the space of gene scores for all marker genes across all groups. Hierarchical clustering is performed within Morpheus (https://software.broadinstitute.org/morpheus) across all rows and columns using one minus the Pearson correlation for the distance metric.

### Constellation plots

To visualize and quantify the global relationships and connectedness between cell types, cell type subclusters, or cell type-area groups, we implemented the constellation plots described in ref. ^1^, by adapting the code made available at https://github.com/AllenInstitute/scrattch.hicat/. In brief, we represented each group of cells as a node, whose size is proportional to the number of cells contained within it. Each node is positioned at the centroid of the UMAP coordinates of its cells. Edges represent relationships between nodes, and were calculated by obtaining the 15 nearest neighbours for each cell in principal component analysis space (principal components 1:50), then determining, at each cluster, the fraction of neighbours belonging to a different cluster. An edge is drawn between 2 nodes if >5% of nearest neighbours belong to the opposite cluster in at least one of them. An edge’s width at the node is proportional to the fraction of nearest neighbours belonging to the opposite node, with the maximum fraction of out-of-node neighbours across all clusters represented as an edge width of 100% and equal to node width. The full code adaptation and implementation of this analysis is described in the function buildConstellationPlot in this paper’s associated Github repository.

### Quantification of constellation plots

Constellation plots were quantified by using a summary of the input values described above. For each cell type or area connection, the number of edges between two groups was multiplied by the average fraction of cells meeting the threshold for a connection within the group. This resulting number was called the connectivity index.

### Module eigengene calculations

Module eigengenes were calculated for numerous gene sets using the the R package WGCNA^30^. Scores were generated for each set of up to 10,000 randomly subsetted cells from the group using the function moduleEigengene function, Scores were calculated based on the intersection of the gene set of interest and genes expressed in the subset of cells. For the area-specific signatures, differential expression was performed as described above, and the gene signatures from late stage neurons across all areas were used to calculate module eigengenes for the radial glia and IPC populations.

### Area-specific markers and gene score calculations

The expression profiles of cells from each subcluster or cortical area were compared to those of all other cells using the two-sided Wilcoxon rank-sum test for differential gene expression implemented by the function FindAllMarkers in the R package Seurat and selected based on an adjusted *P*-value cut-off of 0.05. Adjusted *P*-values were based on Bonferroni correction using all features in the dataset. We performed this step separately for each cell type and each individual, since we observed that gene specificity was highly dynamic throughout the developmental process. We then combined the individual gene lists of each cell type and area, and annotated the stage(s) at which each gene appeared to be specific. We binned individuals into three stages: early (GW14, GW16 and GW17), middle (GW18, GW19 and GW20) and late (GW22 and GW25). We ranked upregulated genes by specificity by calculating their gene score, which we defined as the result of a gene’s average log fold-change × enrichment ratio, in turn defined as the percentage of cells expressing the gene in the cluster of interest divide by the percentage of cells expressing in the complement of all cells. Dot plots used to visualize the expression of distinct marker genes across cell types and/or cortical areas were generated the custom function makeDotPlot available in our code repository, which makes use of the Seurat function DotPlot. In brief, for each gene, the average expression value of all non-zero cells from each group (cortical area) is scaled using the base R function scale(), yielding *z*-scores. Scaling is done to enable the visualization of genes across vastly different expression ranges on the same colour scale.

### Transcription factor annotation

Areally enriched marker genes obtained as described above were annotated against a comprehensive list of 1,632 human transcription factors described in^31^ and downloaded from the transcription factor database AnimalTFDB3^32^, available at http://bioinfo.life.hust.edu.cn/static/AnimalTFDB3/download/Homo_sapiens_TF.

### Gene signature overlap and Sankey diagrams

To quantify the degree of (dis)similarity of molecular signatures across distinct cell types, cortical areas, and/or developmental stages, we calculated the overlap between all sets of cell type and area-specific gene markers at each stage, and visualized these comparisons using Sankey diagrams using the function ggSankey from the ggvis R package. We then calculated the proportion of genes for each node shared with every other node, and clustered nodes hierarchically by calculating their euclidean distances based on their proportions of shared genes. The code used to construct the overlap matrices, create the plots and quantify the results is described in the functions buildSankey and buildHeatmap in our Github repository.

### RNA velocity

Velocity estimates were calculated using the Python 3 packages Velocyto v0.17^22^ and scVelo v0.2.2^23^. Reads that passed quality control after clustering were used as input for the Velocyto command line implementation. The human expressed repeat annotation file was retrieved from the UCSC genome browser. The genome annotation file used was provided by CellRanger. The output loom files were merged and used in scVelo to estimate velocity. For the combined cortical analysis, cells underwent randomized subsampling (fraction = 0.5), and were filtered based on the following parameters: minimum total counts = 200, minimum spliced counts = 20 and minimum unspliced counts = 10. The final processed object generated a new UMI count matrix of 18,970 genes across 195,775 cells, for which the velocity embedding was estimated using the stochastic model. The embedding was visualized using UMAP of dimension reduction. The velocity genes were matched by cortical area and were estimated using the rank velocity genes function in scVelo. Computational analysis of the transcriptomic data described in detail above were performed using R 4.0^24^ and Python 3, the R packages Seurat (version 2 and version 3)^25,26^, googleVis^27^, dplyr and ggplot2^28^, the Python packages Velocyto v0.17^22^ and scVelo v0.2.2^23^ as well as the custom-built R functions described. Our reproducible code is available in the Github repository associated with this manuscript.

### Validation marker gene selection

Marker genes for validation with the spatial omics platform were chosen first by identify useful cell type markers within the dataset. *SOX2* was chosen to mark radial glia, *EOMES* was chosen to mark IPCs, and *BCL11B* and *SATB2* were chosen to marker excitatory neuronal populations with previously validated changing co-expression patterns. *POLR2A* was used as a positive control for the technology. The remaining genes were selected based upon their status as a specific marker gene for excitatory neuron clusters of interest.

### Rebus Esper spatial ‘omics platform

Samples for spatial transcriptomics were dissected from primary tissue as described above. Samples were flash frozen in OCT following the protocol described in the osmFISH protocol^33^. Samples were then mounted to APS-coated coverslips, and fixed for 10 min in 4% PFA. Samples were then washed with PBS, and processed for spatial analysis. The spatially resolved, multiplexed in situ RNA detection and analysis was performed using the automated Rebus Esper spatial omics platform (Rebus Biosystems). The system integrates synthetic aperture optics (SAO) microscopy^34^, fluidics and image processing software and was used in conjunction with smFISH chemistry. Individual transcripts from target genes were automatically detected, counted, and assigned to individual cells, generating a cell × feature matrix that contains gene-expression and spatial location data for each individual cell, as well as registered imaging data, as follows.

Rebus Biosystems proprietary software was used to design primary target probes (22–96 oligonucleotides) and corresponding unique readout probes (assigned and labelled with Atto dyes) for each gene. The oligonucleotides were purchased from Integrated DNA Technologies and resuspended at 100 µm in TE buffer. Coverslips (24 x 60 mm, no. 1.5, catalogue (cat.) no. 1152460, Azer Scientific) were functionalized as previously published^33^. Fresh frozen brain tissue sections (10 µm) were cut on a cryostat, mounted on the treated coverslips and fixed for 10 min with 4% paraformaldehyde (Alfa Aesar, catalogue no.) in PBS at room temperature, rinsed twice with PBS at room temperature and stored in 70% ethanol at 4 °C before use. The sample section on the coverslip was assembled into a flow cell, which was then loaded onto the instrument. The hybridization cycles and imaging were done automatically under the instrumental control software. In brief, primary probes for all target genes were initially hybridized for 6 h and probes not specifically bound were washed away. Readout probes labelled with Atto532, Atto594 and Atto647N dyes for the first 3 genes were then hybridized, washed, counterstained with DAPI and then imaged with an Andor sCMOS camera (Zyla 4.2 Plus, Oxford Instruments) through a 20×, 0.45 NA dry lens (CFI S Plan Fluor ELWD, Nikon) with a 365-nm LED for DAPI and 532-nm, 595-nm and 647-nm lasers configured for SAO imaging. Multiple fields of view (FOVs) were imaged for each channel within the region of interest (ROI). Single *z*-planes with 2.8 µm depth of field were acquired for each FOV. After imaging, the first three readout probes were stripped and the readout probes for the next three genes were then hybridized, imaged and stripped. This process was repeated until readout was completed for all genes.

Using the Rebus Esper image processing software, the raw images were reconstructed to generate high-resolution images (equivalent or better than images obtained with a 100× oil immersion lens). RNA spots were automatically detected to generate high fidelity RNA spot tables containing *xy* positions and signal intensities. Nuclei segmentation software based on StarDist^35^ identified individual cells by finding nuclear boundaries from DAPI images. The detected RNA spots were then assigned to each cell using maximum distance thresholds. The resulting cell × feature matrix contains gene counts per cell along with annotations for cell location and nuclear size.

### Kernel density estimation plots

Kernel density estimation plots were created from individual gene spot location maps retrieved from the spatial transcriptomics pipeline. They were created using the seaborn kdeplot function in Python with shading and cmap colouring. They were merged together for Fig. 4 with the Adobe Illustrator overlay and darken features, using 50% opacity.

### Spatial co-expression analysis

Using the cell × feature matrices, we eliminated all spots with less than ten counts for signal. Pearson’s correlations were then performed across the genes within each dataset and filtered for self-correlation. Positive control (POL2RA) and non-excitatory neuron cell type markers (*SOX2*, *EOMES* and *DLX6*) were removed from the analysis. Interactions of 0.05 or more were preserved and visualized with Cytoscape v3.8.2 using a force-directed biolayout. Individual nodes were coloured by their colour in the merged image file in Fig. 4b.

### Reporting summary

Further information on research design is available in the Nature Research Reporting Summary linked to this paper.

## Online content

Any methods, additional references, Nature Research reporting summaries, source data, extended data, supplementary information, acknowledgements, peer review information; details of author contributions and competing interests; and statements of data and code availability are available at 10.1038/s41586-021-03910-8.

## Supplementary information

Supplementary Information This file contains the full descriptions for Supplementary Tables 1–16.

Reporting Summary

Supplementary Tables Supplementary Tables 1–16.

## Data Availability

The data analysed in this study were produced through the Brain Initiative Cell Census Network (BICCN: RRID:SCR_015820) and deposited in the NeMO Archive (RRID:SCR_002001). All counts matrices are freely available at https://data.nemoarchive.org/biccn/grant/u01_devhu/kriegstein/transcriptome/scell/10x_v2/human/processed/counts/, and are organized together at https://assets.nemoarchive.org/dat-9jd8xw6. Source data are provided with this paper.

## Source data

Source Data Fig. 4
Source Data Extended Data Fig. 3
Source Data Extended Data Fig. 7

## Code availability

All code and datasets used in this study, along with single-cell and spatial transcriptomics browsers are available at kriegsteinlab.ucsf.edu/datasets/arealization and https://github.com/carmensandoval/singlecell-neocortex-arealization.
