# Supplementary material for: An atlas of cortical arealization identifies dynamic molecular signatures
Source: Nature. 2021 Oct 6;598(7879):200–4. doi: 10.1038/s41586-021-03910-8 (PMC8494648; doi:10.1038/s41586-021-03910-8)
Supplement: Supplementary file 1 — This file contains the full descriptions for Supplementary Tables 1–16. [file 41586_2021_3910_MOESM1_ESM.pdf]

nature portfolio

https://doi.org/10.1038/s41586-021-03910-8

Supplementary information

An atlas of cortical arealization identifies dynamic molecular signatures

In the format provided by the authors and unedited

An Atlas of Cortical Arealization Identifies Dynamic Molecular Signatures

Aparna Bhaduri[1,2,3*#], Carmen Sandoval-Espinosa[1,2*], Marcos Otero-Garcia[4], Irene Oh[4], Raymund Yin[4], Ugomma C. Eze[1,2], Tomasz J. Nowakowski[1,5], Arnold R. Kriegstein[1,2#]

1. **Department of Neurology, University of California, San Francisco (UCSF), San Francisco, CA, USA.**
2. **The Eli and Edythe Broad Center of Regeneration Medicine and Stem Cell Research, University of California, San Francisco (UCSF), San Francisco, CA, USA.**
3. **Current Address: Department of Biological Chemistry, Los Angeles (UCLA), Los Angeles, CA USA.**
4. **Rebus Biosystems, Inc., Santa Clara, CA**
5. **Department of Anatomy, University of California, San Francisco (UCSF), San Francisco, CA, USA.**

***These authors contributed equally.**
#Correspondence should be addressed to ABhaduri@mednet.ucla.edu or Arnold.Kriegstein@ucsf.edu

Supplementary Table Legends

Supplementary Table 1. Table of metadata for all cells from the whole brain analysis.

Supplementary Table 2. Cluster markers for the final iteration of whole brain combined clustering analysis. Markers were calculated using the one-sided Wilcoxon rank sum test. Table shows gene name, p-value, average log2 fold change, the percentage of cells within the cluster expressing the gene (pct.1), the percentage of cells outside the cluster expressing the gene (pct.2), and adjusted p-value. Positive log-fold change values indicate that the feature is more highly expressed in the cluster of interest.

Supplementary Table 3. Region specific marker genes, including those at the cell type specific level. Markers were calculated using the one-sided Wilcoxon rank sum test. Table shows gene name, p-value, average $\log_2$ fold change, the percentage of cells within the cluster expressing the gene (pct.1), the percentage of cells outside the cluster expressing the gene (pct.2), and adjusted p-value. Positive log-fold change values indicate that the feature is more highly expressed in the cluster of interest.

Supplementary Table 4. Cell type specific differential expression across the neocortex, allocortex (hippocampus), and proneocortex (cingulate). Markers were calculated using the one-sided Wilcoxon rank sum test. Table shows gene name, p-value, average $\log_2$ fold change, the percentage of cells within the cluster expressing the gene (pct.1), the percentage of cells outside the cluster expressing the gene (pct.2), and adjusted p-value. Positive log-fold change values indicate that the feature is more highly expressed in the cluster of interest.

Supplementary Table 5. Table of metadata for all neocortex cells.

Supplementary Table 6. Cluster markers for the final iteration of the neocortex combined clustering analysis. Markers were calculated using the one-sided Wilcoxon rank sum test. Table shows gene name, p-value, average $\log_2$ fold change, the percentage of cells within the cluster expressing the gene (pct.1), the percentage of cells outside the cluster expressing the gene (pct.2), and adjusted p-value.

Supplementary Table 7. Broader cell type signatures for radial glia, IPCs, and excitatory neurons used for module eigengene analysis.

Supplementary Table 8. All differentially expressed genes by cell type across all cortical areas. Markers were calculated using the one-sided Wilcoxon rank sum test. Table shows gene name, p-value, average $\log_2$ fold change, the percentage of cells within the cluster expressing the gene (pct.1), the percentage of cells outside the cluster expressing the gene (pct.2), and adjusted p-value.

Supplementary Table 9. CellxFeature matrix for all spots assigned to cells for the PFC slice analysis, includes x-y coordinates of the GW20 tissue.

Supplementary Table 10. CellxFeature matrix for all spots assigned to cells for the somatosensory cortex slice analysis, includes x-y coordinates of the GW20 tissue.

Supplementary Table 11. CellxFeature matrix for all spots assigned to cells for the temporal cortex slice analysis, includes x-y coordinates of the GW20 tissue.

Supplementary Table 12. CellxFeature matrix for all spots assigned to cells for the V1 cortex slice analysis, includes x-y coordinates of the GW20 tissue.

Supplementary Table 13. CellxFeature matrix for all spots assigned to cells for the PFC slice analysis, includes x-y coordinates of the GW16 tissue.

Supplementary Table 14. CellxFeature matrix for all spots assigned to cells for the somatosensory cortex slice analysis, includes x-y coordinates of the GW16 tissue.

Supplementary Table 15. CellxFeature matrix for all spots assigned to cells for the temporal cortex slice analysis, includes x-y coordinates of the GW16 tissue.

Supplementary Table 16. CellxFeature matrix for all spots assigned to cells for the V1 cortex slice analysis, includes x-y coordinates of the GW16 tissue.
