# Supplementary material for: An atlas of cortical arealization identifies dynamic molecular signatures
Source: Nature. 2021 Oct 6;598(7879):200–4. doi: 10.1038/s41586-021-03910-8 (PMC8494648; doi:10.1038/s41586-021-03910-8)
Supplement: Supplementary file 2 — Reporting Summary [file 41586_2021_3910_MOESM2_ESM.pdf]

nature research

Corresponding author(s): Aparna Bhaduri
Arnold R. Kriegstein

Last updated by author(s): Jun 26, 2021

Reporting Summary

Nature Research wishes to improve the reproducibility of the work that we publish. This form provides structure for consistency and transparency in reporting. For further information on Nature Research policies, see our Editorial Policies and the Editorial Policy Checklist.

Statistics

For all statistical analyses, confirm that the following items are present in the figure legend, table legend, main text, or Methods section.

n/a	Confirmed	
☐	☒	The exact sample size (n) for each experimental group/condition, given as a discrete number and unit of measurement
☐	☒	A statement on whether measurements were taken from distinct samples or whether the same sample was measured repeatedly
☐	☒	The statistical test(s) used AND whether they are one- or two-sided<br>*Only common tests should be described solely by name; describe more complex techniques in the Methods section.*
☒	☐	A description of all covariates tested
☐	☒	A description of any assumptions or corrections, such as tests of normality and adjustment for multiple comparisons
☐	☒	A full description of the statistical parameters including central tendency (e.g. means) or other basic estimates (e.g. regression coefficient) AND variation (e.g. standard deviation) or associated estimates of uncertainty (e.g. confidence intervals)
☐	☒	For null hypothesis testing, the test statistic (e.g. F, t, r) with confidence intervals, effect sizes, degrees of freedom and P value noted<br>*Give P values as exact values whenever suitable.*
☒	☐	For Bayesian analysis, information on the choice of priors and Markov chain Monte Carlo settings
☐	☒	For hierarchical and complex designs, identification of the appropriate level for tests and full reporting of outcomes
☐	☒	Estimates of effect sizes (e.g. Cohen's d, Pearson's r), indicating how they were calculated

Our web collection on statistics for biologists contains articles on many of the points above.

Software and code

Policy information about availability of computer code

Data collection

We used Cell Ranger to align and generate raw count matrices.

Data analysis

Single-cell RNAseq:
Single-cell alignment and counts was performed with cellranger v2.0.0. Doublet removal was performed with scrubleet v0.1. Computational analysis of the transcriptomic data were performed using R 4.0 (R Core Team, 2020) and Python 3, the R packages Seurat (Butler et al, 2019) (version 2 and version 3), googleVis (Gessman, 2011), dplyr and ggplot2 (Wickham et al, 2020; Wickham, 2009), the Python packages Velocyto v0.17 (LaManno, 2018) and scVelo v0.2.2 (Bergen, 2020), as well as the custom-built R functions described.
Hierarchical clustering is performed within Morpheus (https://software.broadinstitute.org/morpheus).

Our reproducible code is available in the Github repository associated with this manuscript.

Spatial transcriptomics:
RNA spot detection, thresholding, cell segmentation, and spot assignment were performed with the Rebus Esper imaging processing software (Rebus Biosystems, Inc., Santa Clara, CA).
Kernel density estimation plots were created from individual gene spot location maps using the kdeplot function implemented in the Python library seaborn. KDE plots were merged with Adobe Illustrator's overlay and darken features, using 50% opacity.
To derive gene co-expression networks from the spatial transcriptomics data, Pearson's correlation between each pair of genes was calculated within each dataset and filtered for self-correlation. Interactions of 0.05 or more were preserved and visualized with Cytoscape v3.8.2 using a force-directed layout.

For manuscripts utilizing custom algorithms or software that are central to the research but not yet described in published literature, software must be made available to editors and reviewers. We strongly encourage code deposition in a community repository (e.g. GitHub). See the Nature Research guidelines for submitting code & software for further information.

Data

Policy information about availability of data

All manuscripts must include a data availability statement. This statement should provide the following information, where applicable:

- Accession codes, unique identifiers, or web links for publicly available datasets
- A list of figures that have associated raw data
- A description of any restrictions on data availability

Single-cell RNA sequencing data has been deposited at the NeMO archive under dbGAP restricted access (RRID:SCR_002001) and metadata for all cells is included in STables 1-2). Counts tables are provided by NeMO without restriction at https://data.nemoarchive.org/biccn/grant/u01_devhu/kriegstein/transcriptome/scell/10x_v2/human/processed/counts/, but raw data is protected to ensure patient privacy.
Spatial transcriptomic data are available for exploration and analysis in STables 9 - 12.

Field-specific reporting

Please select the one below that is the best fit for your research. If you are not sure, read the appropriate sections before making your selection.

☒ Life sciences ☐ Behavioural & social sciences ☐ Ecological, evolutionary & environmental sciences

For a reference copy of the document with all sections, see nature.com/documents/nr-reporting-summary-flat.pdf

Life sciences study design

All studies must disclose on these points even when the disclosure is negative.

Sample size	Because of the scarcity of intact second trimester human samples, we included as many samples per age range as possible. This resulted in at least 3 samples per age range. Sample size was determined by sample availability of rare samples, no sample calculation analysis was performed. However, these samples were sufficient because 3 per age were obtained and all expected major cell types were identified.
Data exclusions	In all the single-cell analyses, we excluded all cells that had fewer than 750 genes per cell and had greater than 10% mitochondrial content. However, these cells re available in the raw dataset. Exclusion criteria were pre-determined based upon published metrics from other single-cell studies.
Replication	All smFISH primary human data had one replicate per area due to the scarcity of the tissue. Replicates for single-cell analysis included at least 3 samples per age range studied. All attempts at replication were successful.
Randomization	No randomization was used. Covariates were not controlled and are not relevant to this study because no covariates were statistically analyzed.
Blinding	No blinding was used. Blinding was not used because all analysis was performed on all relevant samples in reproducible ways so no bias could have been removed by blinding.

Reporting for specific materials, systems and methods

We require information from authors about some types of materials, experimental systems and methods used in many studies. Here, indicate whether each material, system or method listed is relevant to your study. If you are not sure if a list item applies to your research, read the appropriate section before selecting a response.

Materials & experimental systems

n/a	Involved in the study	
☒	☐	Antibodies
☒	☐	Eukaryotic cell lines
☒	☐	Palaeontology and archaeology
☒	☐	Animals and other organisms
☐	☒	Human research participants
☒	☐	Clinical data
☒	☐	Dual use research of concern

Methods

n/a	Involved in the study	
☒	☐	ChIP-seq
☒	☐	Flow cytometry
☒	☐	MRI-based neuroimaging

Human research participants

Policy information about studies involving human research participants

Population characteristics	13 intact deidentified human brain tissue samples from second-trimester specimens spanning gestational weeks 14 to 25. We microdissected 10 major brain structures as available: neocortex, proneocortex (cingulate), allocortex (hippocampus),

	claustrum, ganglionic eminences (GE), hypothalamus, midbrain, striatum, thalamus and cerebellum. No demographic information was collected, including gender, genotype or other treatment history and thus was not analyzed in any co-variate manner.
Recruitment	De-identified tissue samples were collected with previous patient consent in strict observance of the legal and institutional ethical regulations. This was performed by the clinic and no recruitment criteria were used. Because we have no demographic information about either our samples or the patient population, we cannot comment on how any bias may or may not be present. While this would be scientifically helpful, this wall is essential for the patient confidentiality of sensitive procedures and patient privacy trumps our interest.
Ethics oversight	Human Gamete, Embryo, and Stem Cell Research Committee (Institutional Review Board) at the University of California, San Francisco.

Note that full information on the approval of the study protocol must also be provided in the manuscript.
